# Phytogenic Water Additives Improve Broiler Growth Performance via Modulation of Intermediary Metabolism-Related Signaling Pathways

**DOI:** 10.3390/ani11030750

**Published:** 2021-03-09

**Authors:** Joshua J. Flees, Nima K. Emami, Elizabeth Greene, Bhaskar Ganguly, Sami Dridi

**Affiliations:** 1 Center of Excellence for Poultry Science, University of Arkansas, Fayetteville, AR 72701, USA; jjf0021@auburn.edu (J.J.F.); nkhodamb@uark.edu (N.K.E.); esgreene@uark.edu (E.G.); 2 Clinical Research, Ayurvet Limited, Baddi, Himachal Pradesh 173205, India; clinical01@ayurvet.in

**Keywords:** phytogenic water additives, lipogenesis, lipolysis, protein synthesis, broiler chickens

## Abstract

**Simple Summary:**

Global concern around and heightened sensitivity to emerging drug-resistant superbugs have energized scientists to search for new alternatives to in-feed antibiotics. Powered by consumer demand for natural products and due to their beneficial effects on growth performances, phytogenics have become very popular and favorable alternatives. However, their mode of action has not been fully defined. Here, we showed that supplementation of phytogenics (AVSSL and SG) in water modulates peripheral metabolic pathways (reduction in hepatic fatty acid synthesis, mobilization of fat stores, and enhancement of muscle protein synthesis), which might explain, at least partly, their effect on feed efficiency improvement in broilers.

**Abstract:**

A ban on the use of antibiotic growth promoters (AGPs) has fueled and promoted scientific research towards the identification of reliable and effective alternatives. The supplementation of phytogenics AV/SSL12 (AVSSL) and Superliv Gold (SG) in water has been shown to improve broiler feed efficiency (FE) via modulation of hypothalamic neuropeptides. However, their effects on peripheral metabolic pathways are still unknown. The present study was undertaken to determine the effects of AVSSL and SG on lipid and protein metabolism-associated pathways in various tissues. Day-old male Cobb 500 chicks (*n* = 288) were randomly assigned to 3 treatment groups, with 8 replicates of 12 birds each. The treatment groups were fed a basal diet and supplemented with AVSSL or SG in the drinking water at a rate of 2, 4, and 7 mL/100 birds/d during the starter, grower, and finisher phases, respectively. The control group were fed a basal diet with no additive supplementation. On d 35, liver, adipose, and muscle tissue were collected from one bird per pen (8 birds/group). Data were analyzed using Student’s T-test to compare one treatment group to the control using Graph Pad Prism version 6.0 for Windows. In the liver, the levels of phosphorylated acetyl-CoA carboxylase alpha (ACCα) were significantly increased in both the AVSSL and SG groups compared to the control. The hepatic expression of sterol regulatory element-binding protein cleavage-activating protein (SCAP) was significantly downregulated in both treated groups compared to the control. AVSSL supplementation downregulated the hepatic expression of SREBP-2 and adiponectin (AdipoQ), while SG administration upregulated hepatic AdipoR1/R2 mRNA abundances compared to the untreated group. Both AVSSL and SG treatments upregulated hepatic stearoyl-CoA desaturase-1 (SCD-1) gene expression compared to their untreated counterparts. In the adipose tissue, the levels of phosphorylated hormone-sensitive lipase (HSL) at Ser855/554 site were increased in both the AVSSL and SG groups compared to the control. However, ATGL protein expression was decreased in SG compared to the untreated group. In the muscle, the levels of phosphorylated mechanistic target of rapamycin (mTOR) were increased in the AVSSL, but decreased in the SG group compared to the control. Collectively, these data indicate that supplementation of the phytogenics AVSSL and SG in water reduced hepatic lipogenesis-related proteins and increased adipose tissue lipolysis- and muscle protein synthesis-associated targets, which might explain, at least partially, the improvement in FE observed in previous research.

## 1. Introduction

A ban on the use of antibiotic growth promoters (AGPs) has energized scientific research to identify reliable and effective alternatives. Probiotics, prebiotics, synbiotics, organic acids, antimicrobial peptides, and phytogenics are among the various groups of alternatives to in-feed antibiotics that have gained popularity in animal production research [1,2,3,4]. Phytogenics, also called phytobiotics or botanicals, are a growing class of in-feed products derived from plants and plant products such as herbs, spices, essential oils, and blends of these products. Phytogenic additives have been well reviewed and reported to have beneficial effects such as improvement in growth performance [5,6,7,8]. In addition to improvement in zootechnical parameters, phytogenic additives were found to have additional beneficial effects including antimicrobial and antioxidant activity [9,10], enhancement of gut integrity and intestinal barrier function [7,11,12]), and an increase in nutrient digestibility [13]. Despite several published papers regarding the effect of phytogenic additives in poultry, the molecular mechanisms through which these additives exert their positive effects are not well defined. 

Previously, our research group showed that supplementation of two phytogenic additives in the drinking water of broilers significantly improved feed conversion ratio by increasing body weight gain without changes in feed intake compared to the control group [14]. The same additives increased slaughter weights, hot carcass weights, cold carcass weights, and breast meat yield, while decreasing relative fat weight compared to the control un-supplemented group [15]. The expression of feeding-related hypothalamic neuropeptides was investigated [14] and previous data showed that these phytogenics modulates the hypothalamic expression of (an)orexigenic neuropeptides [14]. However, the underlying peripheral molecular pathways remained unknown. To gain further insights and to better understand the changes in the weight of adipose tissue and muscle yield, this study was, therefore, undertaken to determine the effect of the two phytogenic water additives (PWAs) on the intermediary metabolism in three metabolically important tissues (liver and adipose tissue for lipogenesis and lipolysis, respectively) and muscle (for protein synthesis). 

## 2. Materials and Methods

### 2.1. Ethical Statement

All animal experiments were approved by the University of Arkansas Institutional Animal Care and Use Committee (IACUC # 16084) and were in accordance with the recommendations in the NIH’s *Guide for the Care and Use of Laboratory Animals*. 

### 2.2. Animal Husbandry and Experimental Design

All animal husbandry and experimental design methods were previously described by our group [14,15]. Briefly, day-old, male broiler chicks (Cobb 500, Cobb-Vantress Inc., Siloam Springs, AR, USA, *n* = 288) were neck tagged, individually weighed, and randomly allotted into 24 floor pens. Each pen received fresh pine shavings, and was equipped with a separate feeder (Choretime feeders; Georgia Poultry, Newton Grove, NC, USA) and supplemental waterer. The 24 pens were assigned to 3 treatment groups (8 pens/treatment; 12 birds/pen) that were all fed the same basal starter, grower, and finisher diets. Treatments include AVSSL or SG supplemented in the drinking water at a rate of 2, 4, and 7 mL/100 birds/d during the starter, grower, and finisher periods, respectively, and a control group with no additive in the water. The formulations for the starter, grower, and finisher diets were published previously [15]. The PWAs were supplied by Ayurvet Ltd. (Kaushambi, Ghaziabad, India) with the composition of the additives being proprietary, but are polyherbal formulations of pre-standardized, tested herbs. All animals were reared according to the Cobb-Vantress Broiler Guidelines until the end of the study. On d 35, one bird per pen was randomly selected, and euthanized by cervical dislocation for the collection of liver tissue from the caudal region of the left lobe, subcutaneous adipose tissue, and *Pectoralis major* (breast) muscle tissue from the left breast. All tissue samples were snap frozen in liquid nitrogen and stored at −80 °C until further analysis. 

### 2.3. RNA Isolation and RT-qPCR

Total RNAs were extracted from the liver, adipose tissue, and muscle samples using Trizol reagent (ThermoFishcher Scientific, Rockford, IL, USA) according to the manufacturer’s recommendations. RNA integrity and quality were assessed using 1% agarose gel electrophoresis. The concentration and purity of the RNA were determined for each sample by Take 3 Micro-Volume Plate using a Synergy HT multi-mode micro plate reader. The RNA samples were RQ1 RNase-free DNase treated (Promega, WI) and RNA (1 μg) were reverse transcribed using a qScript cDNA Synthesis Kit (Quanta Biosciences, Gaithersburg, MD, USA). The reverse-transcribed products (cDNA) were amplified by real-time quantitative PCR (Applied Biosystems 7500 Real-Time PCR system) by using 5 μL of 10x diluted cDNA with a SYBR Green Master Mix (ThermoFisher Scientific, Rockford, IL, USA) combined with 0.5 μM of each forward- and reverse-specific primer in a total of 20 μL reaction in duplicate [16,17]. Oligonucleotide primers specific for chicken ATP citrate lyase (ACLY), acetyl coenzyme A carboxylase alpha (ACCα), fatty acid synthase (FASN), stearoyl coenzyme A desaturase-1 (SCD-1), malic enzyme (ME), sterol regulatory element-binding protein 1/2 (SREBP1/2), SREBP cleavage-activating protein (SCAP), insulin-induced gene 2 (INSIG2), peroxisome proliferator-activated receptor alpha/gamma (PPARα/γ), fatty acid translocase (CD36), adiponectin (AdipoQ), adiponectin receptor 1/2 (AdipoR1/2), and visfatin (NAMPT) were measured in the liver samples. Hormone-sensitive lipase (HSL) was measured in the adipose tissue samples. Lipoprotein lipase (LPL), lipase C (LIPC), and adipose triglyceride lipase (ATGL) genes were measured in liver and adipose tissue samples. Finally, mechanistic target of rapamycin (mTOR), ribosomal protein S6 kinase beta-1 (RPS6KB1), adenosine monophosphate-activated protein kinase alpha 1/2 (AMPKα1/2), AMPK beta 1/2 (AMPKβ1/2), and AMPK gamma 1–3 (AMPKγ1–3) were measured in the muscle samples. The ribosomal 18S subunit gene was utilized as the housekeeping gene for analysis. The oligonucleotide primer sequences have been previously reported [16,18,19,20]. The cycling conditions for RT-qPCR were 50 °C for 2 min, 95 °C for 10 min, and 40 cycles of a two-step amplification process (95 °C for 15 s and 58 °C for 1 min). At the end of the amplification process, a melting curve analysis was applied using the dissociation protocol for the Sequence Detection System to exclude contamination with unspecific PCR products (Lassiter et al., 2015). The relative expression of target genes was normalized to the expression of 18S rRNA and calculated using the 2^–ΔΔCt^ method [21] with the control group as the calibrator.

### 2.4. Western Blot Analysis

Protein extractions from the abovementioned tissues were performed using lysis buffer and homogenization methods for Western blot analysis that were previously described [16]. Protein concentrations were determined using a Bradford assay kit (BioRad, Hercules, CA) with bovine serum albumin as a standard curve using a Synergy HT multi-mode microplate reader. Once protein concentrations were determined, LDS sample buffer was added to 80 µg of each protein sample, heated at 70 °C for 10 min, and vortexed before gel electrophoresis in 4–12% gradient Bis-Tris gels (Life Technologies, Waltham, MA, USA). Gel electrophoresis was conducted at 120 V for 60 min with NuPage MOPS running buffer (Life Technologies, Waltham, MA, USA). Gels were transferred to polyvinylidene difluoride (PVDF) membranes in an Invitrogen system using NuPage transfer buffer (Life Technologies, Waltham, MA, USA) at 100 V for 75 min. Transferred membranes were blocked for 60 min in a solution of 5% non-fat dry milk dissolved in Tris-buffered saline plus 0.01% Tween 20 (TBS-T). After blocking, membranes were washed in TBS-T twice for 15 min. Blocked membranes were incubated with a primary antibody diluted to 1/1000 overnight at 4 °C. The following polyclonal antibodies were used: rabbit anti-FASN (Novus Biologicals, Littleton, CO, USA), rabbit anti-phospho ACC (Cell Signaling Technologies, Danvers, MA, USA), rabbit anti-ACC, rabbit anti-ACLY (LSBio, Seattle, WA, USA), rabbit anti-ME (Cell Signaling Technologies, Danvers, MA), rabbit anti-ATGL (Cell Signaling Technologies, Danvers, MA, USA), rabbit anti-phospho HSL^Ser 855/554^ (Cell Signaling Technologies, Danvers, MA, USA), rabbit anti-HSL (Cell Signaling Technologies, Danvers, MA, USA), rabbit anti-phospho mTOR ^Ser 2481^ (Cell Signaling Technologies, Danvers, MA, USA), and rabbit anti-mTOR (Cell Signaling Technologies, Danvers, MA, USA). Rabbit anti-GAPDH (Cell Signaling Technologies, Danvers, MA, USA), rabbit anti-β-actin (Cell Signaling Technologies, Danvers, MA), and rabbit anti-vinculin served as housekeeping proteins. The secondary horseradish peroxidase-conjugated antibody (Santa Cruz Biotechnology, Dallas, TX, USA), at a dilution of 1:5000, was used and incubated for 60 min at room temperature. A pre-stained molecular weight marker (Precision Plus Protein Dual Color) was used as a standard (BioRad, Hercules, CA, USA). The signal was visualized by enhanced chemiluminescence (ECL plus; GE Healthcare Bio-Sciences, Buckinghamshire, UK) and captured by the FluorChem M MultiFluor System (Proteinsimple, Santa Clara, CA, USA). Image acquisition and analysis were performed using AlphaView software (Version 3.4.0, 1993–2011, Proteinsimple, Santa Clara, CA, USA).

### 2.5. Statistical Analysis

Gene and protein expression data were analyzed using Student’s T-test to compare one treatment group to the control using Graph Pad Prism version 6.0 for Windows (Graph Pad Software, La Jolla, CA, USA). Differences were considered significant at *p* ≤ 0.05.

## 3. Results

### 3.1. Hepatic Lipogenesis-Related Pathways

**Figure 1:**
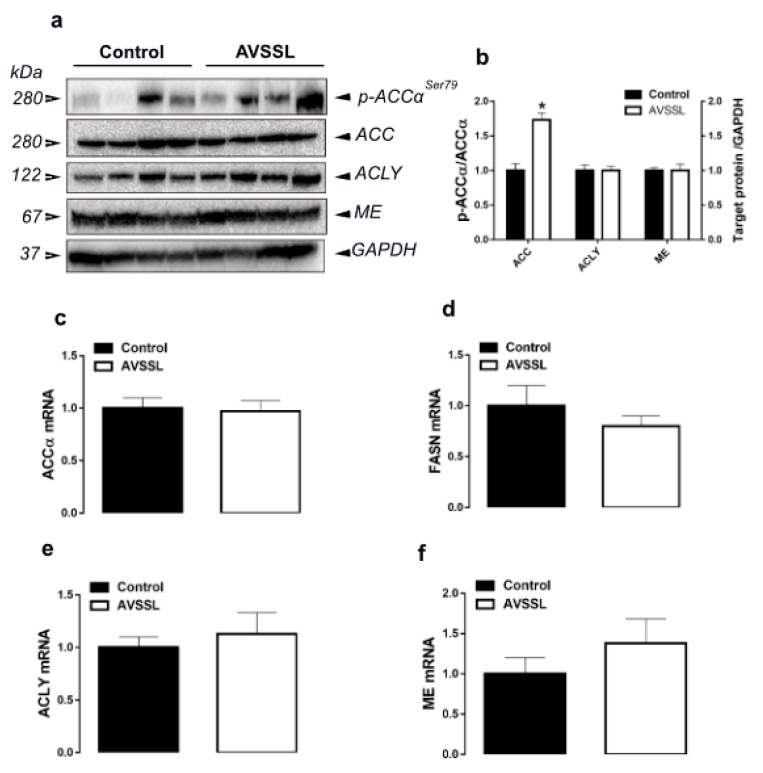
Effect of the phytogenic water additive AVSSL on the expression of lipogenesis-related proteins in liver tissue. Protein levels (**a**,**b**) were measured by Western blot and mRNA abundances (**c**–**f**) were determined by qPCR using the 2^− ∆∆Ct^ method [21]. Data are presented as the mean ± SEM (*n* = 8 birds/ group). * Denotes significant difference compared to the control group at *p* < 0.05. ACCα, phosphorylated-acetyl CoA carboxylase; ACLY, ATP citrate lyase; FASN, fatty acid synthase; ME, malic enzyme.

**Figure 2:**
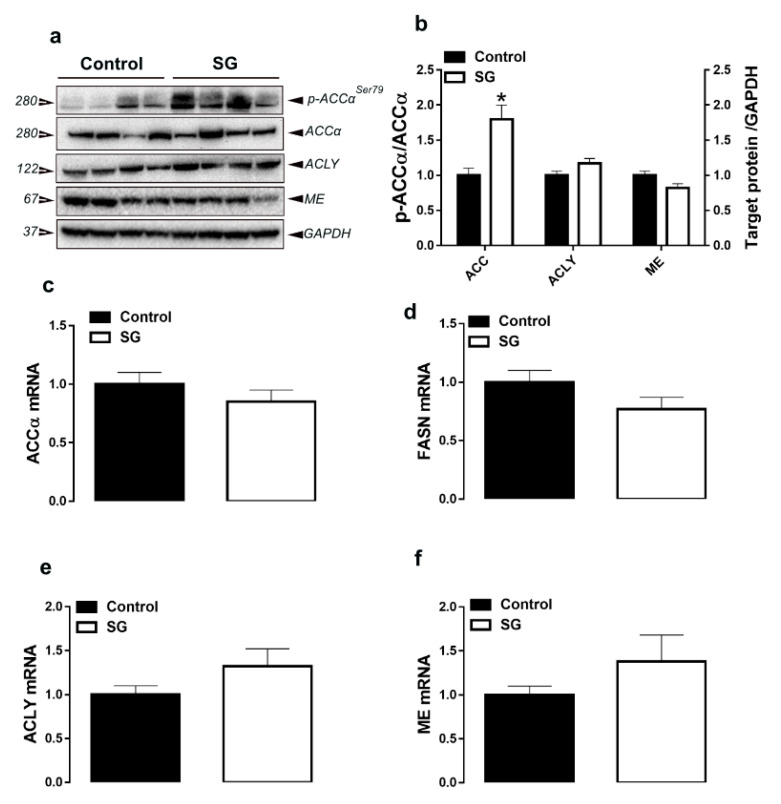
Effect of the phytogenic water additive SG on the expression of lipogenesis-related proteins in liver tissue. Protein levels (**a**,**b**) were measured by Western blot and mRNA abundances (**c**–**f**) were determined by qPCR using the 2^−∆∆Ct^ method [21]. Data are presented as the mean ± SEM (*n* = 8 birds/ group). * Denotes significant difference compared to the control group at *p* < 0.05. ACCα, phosphorylated-acetyl CoA carboxylase; ACLY, ATP citrate lyase; FASN, fatty acid synthase; ME, malic enzyme.

Supplementation of AVSSL and SG deactivated ACCα via increasing the levels of phosphorylated ACCα at Ser79 site compared to the control (Figure 1a,b and Figure 2a,b). Both phytogenics significantly upregulated hepatic SCD-1 gene expression, but without eliciting any changes to FASN, ACLY, ME gene and protein expressions (Table 1 and Table 2; Figure 1c–f and Figure 2c–f). For the transcription factors, there was a significant decrease in hepatic SREBP-2 mRNA levels in the AVSSL-treated group, and hepatic SCAP mRNA abundances in both treated groups compared to the untreated counterpart (Table 1 and Table 2). The hepatic expression of SREBP-1, PPARα, PPARγ, and INSIG2 remained unchanged between all the groups (Table 1 and Table 2).

AVSSL administration significantly downregulated the hepatic expression of AdipoQ compared to the control (Table 1), without affecting CD36, AdipoR1/2 and NAMPT (Table 1). SG treatment, on the other hand, significantly upregulated the hepatic expression of AdipoR1/2 genes, without any changes to CD36, AdipoQ or NAMPT gene expression compared to the control group (Table 2).

### 3.2. Lipolysis-Related Genes and Proteins in Adipose Tissue

**Figure 3:**
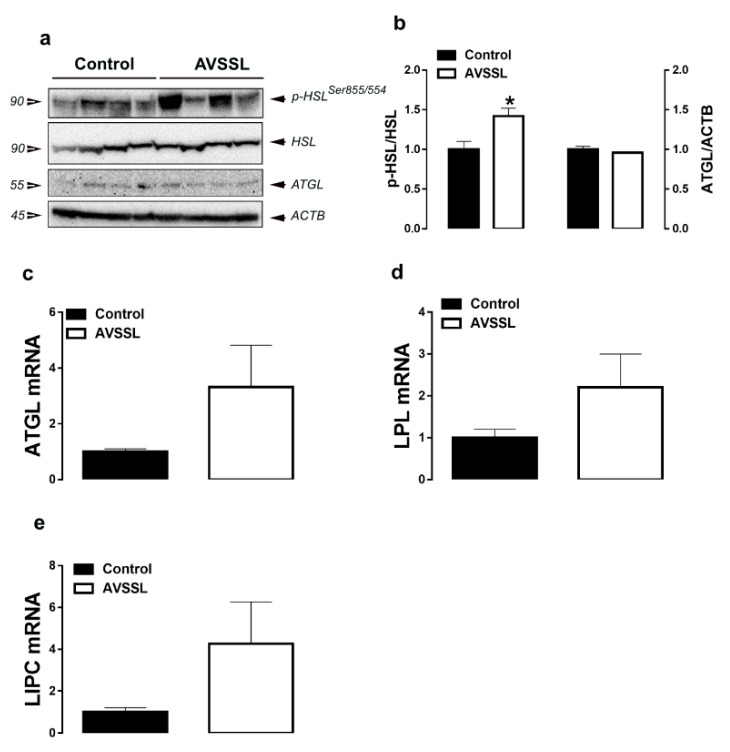
Effect of the phytogenic water additive AVSSL on the expression of lipolysis-related proteins and genes in adipose tissue. Protein levels (**a**,**b**) were measured by Western blot and mRNA abundances (**c**–**e**) were determined by qPCR using the 2^−∆∆Ct^ method [21]. Data are presented as the mean ± SEM (*n* = 8 birds/ group). * Denotes significant difference compared to the control group at *p* < 0.05. ATGL, adipose triglyceride lipase; HSL, hormone-sensitive lipase; LPL, lipoprotein lipase; LIPC, lipase C.

**Figure 4:**
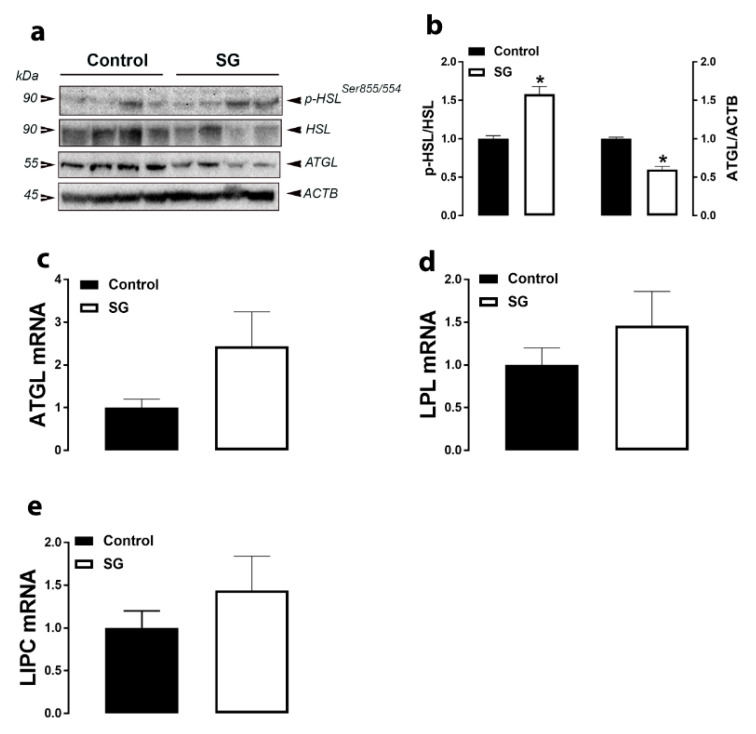
Effect of SG on the expression of lipolysis-related proteins and genes in adipose tissue. Protein levels (**a**,**b**) were measured by Western blot and mRNA abundances (**c**–**e**) were determined by qPCR using the 2^−∆∆Ct^ method [21]. Data are presented as the mean ± SEM (*n* = 8 birds/ group). * Denotes significant difference compared to the control group at *p* < 0.05. ATGL, adipose triglyceride lipase; HSL, hormone-sensitive lipase; LPL, lipoprotein lipase; LIPC, lipase C.

Supplementation of both AVSSL and SG into the drinking water significantly increased adipose tissue p-HSL^Ser855/554^ levels compared to the control group (Figure 3a,b and Figure 4a,b). SG treatment, but not AVSSL, reduced ATGL protein levels compared to the control group (Figure 3a,b and Figure 4a,b). Adipose tissue LIPC, LPL, and ATGL mRNA abundances were not affected by all treatments (Figure 3c–e and Figure 4c–e ).

### 3.3. Protein Synthesis-Related Pathway in Breast Muscle 

**Figure 5:**
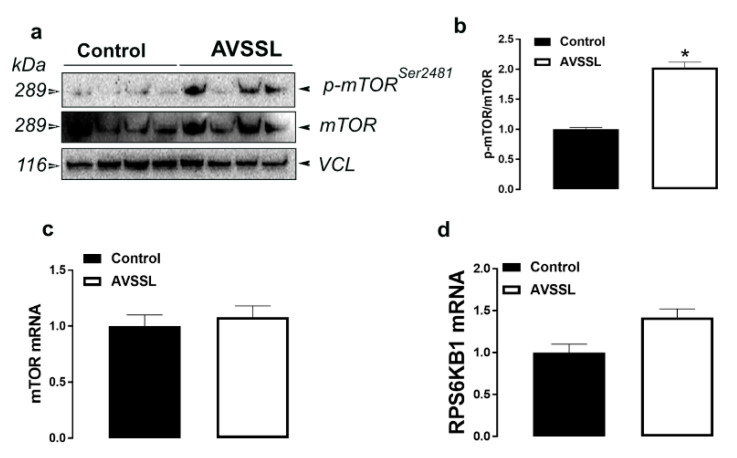
Effect of the phytogenic water additive AVSSL on the expression of protein metabolism-associated proteins and genes in breast muscle tissue. Protein levels of phosphorylated mTOR, mTOR, and VCL (**a**,**b**) were measured by Western blot and mRNA abundances of mTOR and RPS6KB1 (**c**,**d**) were determined by qPCR using the 2^−∆∆Ct^ method [21]. Data are presented as the mean ± SEM (*n* = 8 birds/ group). * Denotes significant difference compared to the control group at *p* < 0.05. mTOR, mechanistic target of rapamycin; RPS6KB1, ribosomal protein S6 kinase beta-1.

**Figure 6:**
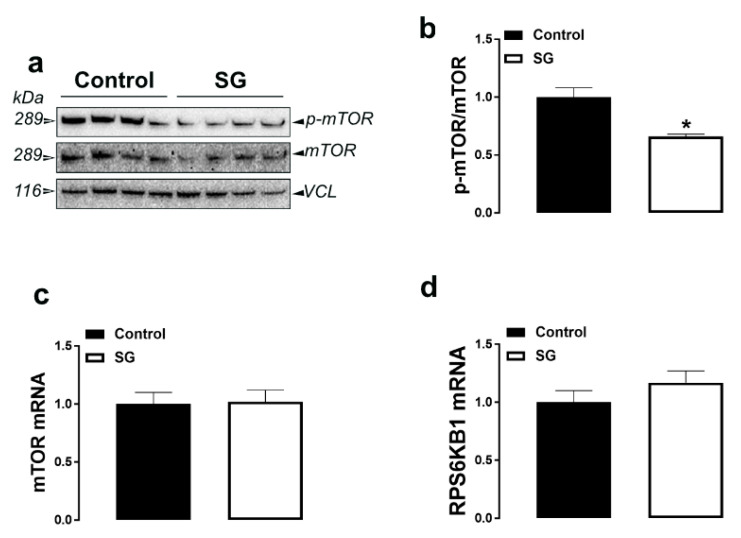
Effect of the phytogenic water additive SG on the expression of protein metabolism-related proteins and genes in breast muscle tissue. Protein levels of phosphorylated mTOR, mTOR, and VCL (**a**,**b**) were measured by Western blot and mRNA abundances of mTOR and RPS6KB1 (**c**,**d**) were determined by qPCR using the 2^−∆∆Ct^ method [21]. Data are presented as the mean ± SEM (*n* = 8 birds/ group). * Denotes significant difference compared to the control group at *p* < 0.05. mTOR, mechanistic target of rapamycin; RPS6KB1, ribosomal protein S6 kinase beta-1.

The levels of phosphorylated mTOR at Serine 2481 site were significantly increased in the breast muscle of AVSSL-treated birds, and decreased in SG-supplemented groups compared to the control (Figure 5a,b and Figure 6a,b). Muscle mTOR, RPS6KB1, AMPKα1/2, AMPKβ1/2, and AMPKγ1–3 mRNA abundances remained unchanged between all groups (Figure 3 and Figure 4 and Table 3 and Table 4).

## 4. Discussion

Published data from our research group showed that supplementation of AVSSL and SG to the drinking water of broilers improved feed efficiency (increased body weight without changing feed intake compared to the control group), and improved processing parameters such as higher breast meat yields and lower fat pad weights [14,15]. A positive energy balance is a result of complex interactions between energy intake (that is under the control of the hypothalamus), energy expenditure and peripheral intermediary metabolism. In a previous report [14], we studied the role of the hypothalamic neuronal circuits and showed that AVSSL and SG modulate the expression of feeding-related hypothalamic neuropeptides. To gain further insights and by using three metabolically important tissues, the objective of the present study was to identify the peripheral molecular mechanisms that are responsible for the positive impacts of AVSSL and SG on broiler performance. 

The chicken liver is the main site of *de novo* lipogenesis [22]. It has been reported that more than 90% of *de novo* synthesis of fatty acids occurs in the chicken liver [23,24]. Lipogenesis is controlled by several key enzymes, including ACCα, which catalyzes the carboxylation of acetyl-CoA to malonyl-CoA, serving as a rate-limiting enzyme in fatty acid biosynthesis [25,26]. ACCα is inactivated by phosphorylation at the serine 79 site [27]. Supplementation of AVSSL and SG in the drinking water increased the expression of pACCα, which might explain previously observed decrease in fat pad weights in both the AVSSl- and SG-treated groups compared to the control [15]. This is further supported by the downregulation of SCAP gene in both the AVSSL and SG groups, as SCAP serves as a key enzyme in activating SREBP-1, which is a key transcription factor for lipogenesis. Knockdown of SCAP in the liver tissue of rhesus monkeys has been shown to decrease *de novo* lipogenesis [28].

Intriguingly, the differential modulation of hepatic adiponectin system by the two phytogenics (downregulation of AdipoQ by AVSSL and upregulation of AdipoR1/2 by SG) is puzzling. Although AdipoQ is highly expressed in the chicken liver [29], its role is not well established. However, AdipoQ expression in adipose tissue has been shown to be inversely related to abdominal fat deposition [30]. Chicken globular adiponectin impairs adipocyte differentiation via p38 MAPK/ATF-2 and mTOR pathways [31], and inhibits lipid deposition in adipocytes by increasing the expression of ATGL. Together, these data suggest that the effects of adiponectin are probably type (globular vs. full-length adiponectin) and tissue specific (adipose tissue vs. liver) and this needs further in-depth investigation. In addition, we measured only the mRNA levels of the adiponectin system, which represents a limitation in this study, and protein levels might be different due to posttranscriptional and translational modification as previously shown [32].

As SCD-1 is a key enzyme in the synthesis of unsaturated fatty acids, principally the omega 9 oleic acid via forming a double bond in stearoyl-CoA and desaturation of stearic acid, its upregulation in our experimental conditions suggests that WPAs modify the fatty acid profile. This may also, at least partially, explain the beneficial effects of WPAs as oleic acids has been reported to mitigate inflammation through reducing pro-inflammatory cytokines production [33,34].

Due to a subtle balance between lipogenesis and lipolysis in regulating lipid metabolism homeostasis, the expression of adipose tissue lipolysis-related genes and proteins were determined in this study. The increased levels of phosphorylated HSL at Ser855/554 site by both phytogenics indicated mobilization of fat stores and might explain the decreased fat pad weight. HSL, previously known as cholesteryl ester hydrolase, is a canonical triglyceride, diacylglycerol, and monoacylglycerol hydrolase [35]. Its overexpression resulted in a lower body weight and fat mass in transgenic mice [36]. Similarly, adenoviral overexpression of HSL reduced liver triglycerides by 40–60% in both ob/ob and high fat-induced obese mice [37].

In the muscle, the activation of mTOR indicates that AVSSL enhances protein synthesis, which explain the higher breast yield [15]. However, the decrease in levels of phosphorylated mTOR in the SG group is intriguing, especially when breast yield is increased [15]. The nutrient master, mTOR, is a serine/threonine kinase responsible for controlling protein synthesis and muscle mass hypertrophy [38]. While both WPAs enhance breast yield [15], the opposite regulation of mTOR suggests that these WPAs might increase protein synthesis via different signaling pathways. In fact, protein synthesis is conventionally divided into three stages, namely initiation, elongation, and termination, and each stage involves a number of protein regulators. In addition, mTOR exists in at least two characteristically distinct complexes: a rapamycin-sensitive mTOR complex 1 (mTORC1) and a rapamycin-insensitive mTOR complex 2 (mTORC2) [39]. It is plausible that the WPAs differentially regulate the protein complex at different stages of protein synthesis and/or mTOR complexes and further studies are warranted. Furthermore, we measured only p-mTOR^Ser2481^ in this study. However, there are several other phosphorylation sites such as T2446, S2448, and S1261, and it is conceivable that AVSSL and SG differentially target these sites. 

## 5. Conclusions

In conclusion, in our experimental conditions, supplementation of AVSSL and SG in drinking water improves FE (increased BW and breast yield, decreased fat content, and without altered feed intake) in broilers via modulation of peripheral intermediary metabolism by enhancing adipose tissue lipolysis and favoring muscle protein synthesis over hepatic lipogenesis.

## Figures and Tables

**Table 1:** Effect of the phytogenic water additive AVSSL on lipid metabolism genes in the liver tissue ^a^.

Parameters ^b^	Experimental Groups ^c^					
	Control			AVSSL		
SCD-1	1.00	±	0.21	1.82	±	0.30 *
SREBP1	1.00	±	0.11	0.79	±	0.10
SREBP2	1.00	±	0.26	0.46	±	0.05 *
SCAP	1.00	±	0.21	0.29	±	0.03 *
PPARα	1.00	±	0.10	1.04	±	0.10
PPARγ	1.00	±	0.14	1.00	±	0.09
INSIG2	1.00	±	0.14	1.00	±	0.14
CD36	1.00	±	0.19	0.86	±	0.18
AdipoQ	1.00	±	0.17	0.61	±	0.08 *
AdipoR1	1.00	±	0.11	1.01	±	0.11
AdipoR2	1.00	±	0.12	1.19	±	0.13
NAMPT	1.00	±	0.11	1.30	±	0.15

^a^ Data are expressed as the mean ± SEM (*n* = 7–8/group). An asterisk (*) denotes a significant difference compared to the control (*p* ≤ 0.05). ^b^ ACCα, acetyl-CoA carboxylase alpha; ACLY, ATP citrate lyase; AdipoQ, adiponectin; AdipoR1/R2, adiponectin receptor 1/2; CD36, fatty acid translocase; FASN, fatty acid synthase; INSIG2, insulin-induced gene; ME, malic enzyme; NAMPT, visfatin; PPAR, peroxisome proliferator-activated receptor; SCAP, sterol regulatory element-binding protein cleavage-activating protein; SCD-1, stearoyl-CoA desaturase; SREBP1/2, sterol regulatory element-binding protein 1/2. ^c^ AVSSL, AVSSL/12.

**Table 2:** Effect of the phytogenic water additive SG on lipid metabolism genes in liver tissue ^a^.

Parameters ^b^	Experimental Groups ^c^					
	Control			SG		
SCD-1	1.00	±	0.21	1.84	±	0.39 *
SREBP1	1.00	±	0.11	0.80	±	0.08
SREBP2	1.00	±	0.26	0.55	±	0.08
SCAP	1.00	±	0.21	0.28	±	0.04 *
PPARα	1.00	±	0.10	1.16	±	0.15
PPARγ	1.00	±	0.14	1.96	±	0.53
INSIG2	1.00	±	0.14	0.90	±	0.16
CD36	1.00	±	0.19	0.72	±	0.15
AdipoQ	1.00	±	0.17	1.48	±	0.34
AdipoR1	1.00	±	0.11	1.52	±	0.21 *
AdipoR2	1.00	±	0.12	1.30	±	0.11 *
NAMPT	1.00	±	0.11	1.43	±	0.25

^a^ Data are expressed as the mean ± SEM (*n* = 7–8/group). An asterisk (*) denotes a significant difference compared to the control (*p* ≤ 0.05). ^b^ ACCα, acetyl-CoA carboxylase alpha; ACLY, ATP citrate lyase; AdipoQ, adiponectin; AdipoR1/R2, adiponectin receptor 1/2; CD36, fatty acid translocase; FASN, fatty acid synthase; INSIG2, insulin-induced gene; ME, malic enzyme; NAMPT, visfatin; PPAR, peroxisome proliferator-activated receptor; SCAP, sterol regulatory element-binding protein cleavage-activating protein; SCD-1, stearoyl-CoA desaturase; SREBP1/2, sterol regulatory element-binding protein 1/2. ^c^ SG, superliv gold.

**Table 3:** Effect of the phytogenic water additive AVSSL on muscle AMPK pathway ^a^.

Parameters ^b^	Experimental Groups ^c^					
	Control			AVSSL		
AMPKα1	1.00	±	0.27	0.97	±	0.10
AMPKα2	1.00	±	0.19	0.79	±	0.19
AMPKβ1	1.00	±	0.05	1.66	±	0.20
AMPKβ2	1.00	±	0.10	0.77	±	0.14
AMPKγ1	1.00	±	0.06	0.94	±	0.11
AMPKγ2	1.00	±	0.18	1.45	±	0.21
AMPKγ3	1.00	±	0.09	0.88	±	0.20

^a^ Data are expressed as the mean ± SEM (*n* = 7–8/group). ^b^ AMPK, adenosine monophosphate-activated protein kinase. ^c^ AVSSL, AVSSl/12.

**Table 4:** Effect of the phytogenic water additive SG on muscle AMPK pathway ^a^.

Parameters ^b^	Experimental Groups ^c^					
	Control			SG		
AMPKα1	1.00	±	0.27	0.93	±	0.31
AMPKα2	1.00	±	0.19	0.93	±	0.15
AMPKβ1	1.00	±	0.05	1.08	±	0.16
AMPKβ2	1.00	±	0.10	0.81	±	0.08
AMPKγ1	1.00	±	0.06	0.89	±	0.05
AMPKγ2	1.00	±	0.18	1.10	±	0.13
AMPKγ3	1.00	±	0.09	0.90	±	0.14

^a^ Data are expressed as the mean ± SEM (*n* = 7–8/group). ^b^ AMPK, adenosine monophosphate-activated protein kinase. ^c^ SG, superliv gold.

## Data Availability

Data is contained within the article.
